# Factors predicting companies’ crisis in the engineering industry from the point of view of financial analysis

**DOI:** 10.1371/journal.pone.0264016

**Published:** 2022-02-18

**Authors:** Jozef Lukáč, Cecília Olexová, Zuzana Kudlová

**Affiliations:** 1 Faculty of Business Economics with seat in Košice, Department of Corporate Financial Management, University of Economics in Bratislava, Bratislava, Slovakia; 2 Faculty of Business Economics with seat in Košice, Department of Management, University of Economics in Bratislava, Bratislava, Slovakia; The Bucharest University of Economic Studies, ROMANIA

## Abstract

A key factor for business management is the assessment of the financial situation of companies. Nowadays, it is essential to monitor the liquidity crisis, which is closely linked to corporate crises. The aim of the paper is to analyse a selected sector of the economy from the perspective of the corporate crisis and to identify the factors of crisis. More than 2000 engineering companies in Slovakia were analysed during the period from 2015 to 2019 with the aim of analysing financial results, especially in the area of financial forecast for the future. In the analysis, statistical testing of the significance of relationships using the Spearman correlation coefficient, the significance of differences by the power of t-test, regression and clustering were used. A significant part of the paper is the analysis of selected indicators of the company’s crisis—Altman’s Z score and the IN05 index. The results indicate that engineering companies in Slovakia are achieving good results and their financial situation is improving within the years between 2015–2019. The results can also be used as a starting point for research concerning the impact of COVID-19 in this area. In the context of corporate crisis management, engineering companies behave in the same way but it is necessary to monitor individual factors that can detect a corporate crisis. Possible measures would thus lead to the stabilization of financial results and long-term sustainable positive prospects for companies in the future.

## Introduction

The Slovak Republic is a highly industrialized country. The Slovak Republic achieves a GDP of which a significant part is made up of the automotive industry—at 49.5%, it has one of the highest rankings in Europe. The industry in Slovakia is dominated mainly by the automotive and engineering sectors, which, together with electrical engineering, are the main sources of growth in industrial production.

Maintaining the continuity of trends is essential to maintain healthy growth and competitiveness when it comes to engineering as well as the whole economy of the Slovak Republic, especially in connection with growing competition from low-cost countries. On the other hand, Slovakia, as open economics is concerned, is an export-oriented type of economy, strongly confronted with the lingering economic recession, which significantly affects the engineering sector in particular and especially when it comes to the motor manufacturing sector of vehicles. Nevertheless, Slovakia manages to keep the prestige of the country attractive when it comes to new investment. The main reasons are mainly the stability of the political environment, favourable tax and labour legislation, qualifications and discipline of the workforce, as well as the still favourable ratio of wages and labour productivity.

The production of motor vehicles, trailers and semitrailers, production of other means of transport and production of machinery not elsewhere classified forming the aggregation of the engineering industry belong to the important segments of the Slovak economy, which also results from the fact that they together provide 35.40% in 2019 share in value added and 30.75% employment in industrial production. Although it should be noted that the Slovak Republic is among the countries within the OECD countries, where the substitution effect on the labour market comparable to other countries is significant [1]. It is estimated that 33% of all jobs can be changed to be highly automated (11% of jobs are directly threatened by automation) and significant changes of job tasks are expected in other jobs (31%), which is more than in many other OECD countries [2]. According to an analysis by [3], automation based on robotics and artificial intelligence is estimated to increase productivity globally by 0.8 to 1.4% per year (scenario for the period 2015–2065).

It is also important that the engineering companies contribute to a higher appreciation of materials from the production of metals, plastics and rubber, although there are some reserves where synergies can be sought, especially in terms of increasing the share of component production in the overall results of the SK NACE 29 sector.

Engineering companies in Slovakia play an important role in job creation, innovation and economic development, also of international importance. Slovakia is a leader in car production in terms of population thanks to the world’s manufacturers VW, Kia Motors, Jaguar Land Rover and Stallantis. A significant company in the engineering industry is also US Steel Košice, which is a subsidiary of the world steel producer—United States Steel Corporation. It should also be noted here that Slovakia has in the past profiled itself as a country with advanced engineering production, as well as in arms production and the current development is a natural outcome of this.

The crisis of companies in the engineering sector caused serious damage to the production activities of many countries and various companies whose financial situation was not so good, which brought them to the brink of bankruptcy. It is necessary for companies to form a methodological approach to assessing the financial crisis of engineering companies, which would become the basis for the application of a certain type of crisis management in domestic engineering companies. Based on such a prediction, we can form and implement a model of financial management evaluation, which will help to determine the need to apply crisis management in the company based on selected financial indicators. An example is from the study of Sylkin et al. [4] in the environment of Ukraine, as a neighbouring country of Slovakia.

From the point of view of the corporate crisis, it is necessary to monitor not only the financial and capital structure or even the internal influences of the company but also the development of the legislative area, which can significantly affect the position of the company when it comes to the context of its financial situation. It is the areas of tax law and new concepts in the economy that can significantly affect its financial situation, taxation, competitiveness or the amount of the tax burden.

The aim of this paper is to analyze the financial results of a sample of more than 2000 engineering companies in Slovakia for the period between 2015 to 2019, in particular to determine whether there are statistically significant differences between financial performance indicators from the perspective of the crisis in companies and the use of these findings as a tool for financial management. The focus of the research is on Slovak companies, as these companies play an important role in the gross national production. The research is targeted particularly on the financial situation of these companies and the dependence among selected financial indicators in a broader context, i.e. the use of financial models as early warning tools for a pending financial crisis.

The rest of this paper is organized as follows: Section 2 provides a brief literature review of the engineering industry in Slovakia and abroad while it also summarizes research hypotheses. Section 3 describes the data, variables and methodology used in the paper. Section 4 presents the main empirical results, especially in the area of financial forecast for the future. The last part is the conclusion.

### Literature review

Bankruptcy prediction is a topic analysed by economists from around the world in recent years. However, opinions on this topic are very similar. Several authors analyse the impact of the crisis in general, others approach the analysis of the industry as a whole and over time the authors also analyse the impact of COVID-19 on the financial situation of companies [5–9]. The key branch of the Slovak economy is engineering. The magazine Engineering dealing with the engineering industry is published in Slovakia, in which the authors [10] describe the events of this branch in Slovakia as well as abroad.

In Slovak geography, the issue of socio-economic transformation and especially the transformation of the industry in the regions is devoted to several important authors from various perspectives. The authors [11], who focused on regions such as Šariš [12], Dolný Spiš [13] and Považie [14], provide a comprehensive view of the differences in development at the regional level during the transformation period. Kováč [15] describes the problem of Slovakia in the field of small and medium-sized enterprises which it can address either by aid integration of domestic producers and support for research for the narrow area in which they do business or support brand new areas that do not yet exist where it is new technologies that need to be developed and production launched that is promising and unoccupied.

As for the engineering industry abroad, several authors deal with it. Among the first German authors was Alban [16] to analyse it in his publication during the period of industrial analysis from 1970 to 1982. Saul [17] dealt with the development of the British industry and foreign competition between 1875 and 1914. Evidence [18] from perhaps, the most successful country of the past 20 years, South Korea, suggests that the learning time in the engineering industry is much longer than anticipated; two decades does not seem to be unusual. It is also argued that due to both the speed of technical change and the globalization of industries, the learning time has been extended in the past several decades. An overview of the development processes in mechanical engineering as well as its importance and structure in comparison with other industries is described in the foreign publication by Murmann [19] as well as the basic processes of thinking and acting in the development and construction of machine systems. Within Industry 4.0, comprehensive digitization offers engineering companies new opportunities to expand their services. Therefore, the authors [20] conducted a case study of TRUMPF, which shows how data became a prerequisite for business model innovation. Few studies focus on the small and medium-sized enterprises (SMEs). Therefore, Schiersch [21] examines the relationship in the German engineering sector using a large and representative data set, in which he finds that small and large companies are on average the most efficient, while medium-sized companies have the most inefficiency. In addition, the analysis found that companies with active owner(s) are significantly more efficient and that capital companies are less efficient than companies with personally responsible owners. Ernst [22] systematically evaluates the patent behaviour of a sample of 50 companies in the German engineering industry, where he analyses the relationship between these patent strategies and the company’s performance. Patent-active companies appear to perform best in the economic performance variables used, where the numbers of international patent applications, the rate of patents valid and highly cited patents were positively related to economic performance. Therefore, the author recommends a differentiated use of patent data. In another paper, the author [23] presents the results of field studies on product development practices in German engineering companies, focusing on managers’ estimates to what extent and by what means they could reduce cycle time and resource utilization if projects were managed differently. Major improvements are among the activities concentrated at the beginning of the development process. The research and development behaviour of industrial firms with LDCs is examined [24], where it analyzes a sample of engineering companies in India to test the hypothesis of technological efforts in LDCs, which is primarily aimed at assimilating and adapting foreign technologies.

The publication of Lall and Kumar [25] examines some of the effects on the export activity of the 100 largest engineering companies in India from 1966 to 1978. In general, the findings illustrate at a disaggregated level the anti-export bias of the industrialization strategy pursued by India. We are currently looking at a much faster change in technology with the necessary accompanying change of skills than ever before. It can be stated that the automotive and engineering industries need the so-called "Skills revolution".

Several publications on the topic of the automotive industry have been made in Slovakia. In their paper, Turisová and Kadárová [26] describe the engineering and automotive industries in Slovakia, which they analyse through traditional indicators (activity, liquidity, profitability) and consider the approach of individual companies, what and how many resources they are able to give up improving their situation in the future. The financial situation of the company reflects the efficiency of the company’s activities, which is determined primarily by the efficiency of production. The existence of a company is determined by the current future financial situation of the company. For this reason, companies are trying to forecast financial health and identify possible financial problems that may arise. The authors [27] emphasize the application of financial models in the conditions of the Slovak Republic and the optimal results of forecasting the financial situation of Slovak companies. Kadárová et al. [28] in the publication Optimal Financing of Industrial Enterprises point out the need to know that there are also new modern ways of financing for small and medium-sized enterprises. The aim of the paper [29] was to monitor the attitudes among industrial enterprises in Slovakia and the Czech Republic for the use and application of the performance management tools. Based on a questionnaire survey in 2006, industrial enterprises in Slovakia and the Czech Republic monitored performance areas management in which companies preferred specific financial indicators and new management tools implemented in 2006. The conclusion of the research pointed to the most watched area–finance, from the point of view in terms of financial indicators such as costs, revenues, profit, liquidity, indebtedness. New trends in performance management which are introduced by businesses are quality management, project management and the financial indicator EVA (venture capital, private capital, etc.), which are little known in Slovakia and remains almost theoretical. Many economists from all over the world have been trying to find a company bankruptcy forecasting model using different methods with the aim to achieve the best results. The authors [30] therefore developed a new modified model that uses regression analysis to obtain higher predictive performance on the analysed sample than selected models. To verify the performance of selected bankruptcy prediction models, they chose an approach based on data mining validation methods.

The analysis of the Slovak automotive industry is examined by Lukáč [31], where he clarifies the possible occurrences of technological effects of spill overs in the Slovak automotive industry for the benefit of domestic companies. Bednárová [32] analyses the development of the automotive industry of the V4 countries in the European automotive market from 2005 to 2016 according to selected criteria, where there is also an evaluation of the position of the automotive industry of the Slovak Republic toward other V4 countries. Several authors have dealt with the development of selected financial ratios in the automotive industry in Slovakia. The analysis of the automotive industry was carried out by the author, [33] who considers it to be a complex sector which primarily affects the areas of engineering, metallurgical, electrical, chemical industry as well as the production of rubber and plastics. The competitiveness of this the industry was also addressed by the authors [34], who described other developments of the automotive industry in the Slovak Republic and selected EU countries. The result is the need to innovate both products and processes, along with restructuring, which is becoming a key requirement for further growth.

In the case of traditional indicators, the authors [35,36] examined profitability [37], liquidity [38,39] and indebtedness [40]. The authors [41] dealt with the issue of bankruptcy models in Slovakia, which is still relevant to high competition in the markets and still problematic. Not only in the world but also in our country, we can see a huge number of bankrupt companies. If a company wants to prosper and compete successfully in a market environment, there should be a regular financial analysis of its activities, the evaluation of successes and failures, results obtained on the basis of a strategic decision on the future of business development. The models for predicting the financial health of the company [42] provide an opportunity to classify the surveyed companies quickly and relatively effectively. In the paper, the authors tested three variants of the Altman model from 2014. They found that the new data gave approximately the same results as the original data only for the third model working with property logarithms. With regard to globalization, microeconomic as well as macroeconomic changes have an immediate impact on individual companies. In earlier periods, Klieštik et al. [43] perceived the ethical aspects of bankruptcy only at the moment of the declaration of bankruptcy. Valášková et al. [44] comprehensively compiled an overview of the possibilities of predicting the company through bankruptcy models. They described and defined each model in detail, described its specifics and described the calculation procedure.

For the purposes of the paper, we have chosen the basic hypotheses from which we will proceed in the analysis of engineering companies, as follows:

*Hypothesis 1*: There are statistically significant differences between the values of indicators of the crisis of engineering companies between the analysed years.

*Alternative hypothesis*: There are no statistically significant differences between the values of the indicators of the crisis of engineering companies between the analysed years.

*Hypothesis 2*: Within the indicators of the corporate crisis—Altman Z-score and the IN05 index, there are statistically significant factors that affect the value of these indicators.

*Alternative hypothesis*: Within the indicators of the corporate crisis—Altman’s Z-score and the IN05 index, there are no statistically significant factors that affect the value of these indicators.

From the above research questions (hypotheses), we can say that the study will focus on the analysis of the engineering sector in Slovakia in terms of the crisis, which is caused by the deterioration of financial results represented by the Z-score index INDEX 05 and EBITDA. The Altman index, used across the countries of the world, is considered to be the most used indicator and its use is in various spheres of business. For the Slovak economy, it is recommended to use the indicator adjusted for the Czech economy, which was supplemented by the share of overdue liabilities in sales because insolvency has a great impact on the company’s finances. Its main advantage is the direct applicability to the Slovak accounting system, which would be a problem with several indicators of forecasting the crisis of companies that are not intended for the Slovak legislative environment or their implementation would record distorted results. INDEX 05, also referred to as the creditor bankruptcy index, has explanatory power of more than 85% for the conditions of Czech and Slovak accounting legislation. The choice of the EBITDA indicator was related to the fact that most of the companies from the engineering industry that we analyzed have business activities in an international environment. The indicator excludes the tax and interest burden and at the same time usually takes into account the main non-monetary costs such as depreciation, which eliminates it. In our terms and conditions, this indicator serves as the "Test of a company in difficulty", on the basis of which the ratio of the company’s profit before interest, tax and depreciation (EBITDA) to interest coverage is determined. If it is less than 1.0 then the company is in crisis and is in difficulty.

## Materials and methods

In our analysis, we used data that represents information from the financial statements of Slovak engineering companies. First of all, it was necessary to select from all companies that we had data for engineering companies in the database. We did not analyse other industries and thus excluded them from the research. The data we had available includes data from the Balance Sheet, the Profit and Loss Statement and the notes to the financial statements. This data is for the period from 2015 to 2019, as a 5-year period is considered to be a general rule to make a sufficient financial analysis. Moreover, we selected these years with an emphasis on comparability, as these years were without other significant external impacts: there was significant change in the distribution of political power at the beginning of this period ensuring stable economic environment (change from left-wing politics to right-wing), constant foreign investment inflow, no exceptional external shocks (e.g. such as the Covid-19 pandemic which is currently ongoing). The branches according to SK NACE which we used in the research can be found in appendix A in S1 Appendix. In the analysis, we used more than 1.700 companies each year.

As part of our contribution, we will use a database of more than 2000 companies in the engineering sector in Slovakia (Appendix A in S1 Appendix) from during the period between 2015 to 2019 (2020 has not yet been reported in the databases due to the postponement of the obligation to file a tax return because of Covid-19). The database contains significant accounting information on the basis of which we were able to determine the selected values of bankruptcy indicators.

The engineering industry in Slovakia is a key area of the economy. This position is based on the historical background and its position within the Slovak economy. The engineering industry is mainly represented by car production and related activities. The engineering industry employs a lot of people not only from Slovakia but also from abroad, namely the countries of the European Union or those in third countries which totalled more than 1.41% in 2018. As part of the analysis of the financial statements of engineering companies in Slovakia, we found that the average number of companies is around 2000. In the engineering sector, no fundamental facts were found that would indicate a rapid decline or increase in the number of companies in this sector. The highest number within the analysed period was in 2017 and conversely, the lowest number of enterprises was recorded in 2015.

For the needs of the analysis of the financial situation of Slovak engineering companies, it was necessary to select key indicators of the financial health of the company. In the financial statements, we focused on indicators that reflect the amount of assets, the balance of receivables and payables, equity, profit before and after taxes as well as sales. From this point of view, we can focus on accounting information starting points that provide research with different starting data than for foreign companies, which are based on different accounting systems. We can say that the results of the crisis indicators will be unique, especially for Slovak and Czech accounting legislation or for similar accounting systems.

To evaluate the selected results of financial analysis, we used statistical methods: statistical testing of the normality of variables, statistical testing of significant relationships between selected indicators using a correlation coefficient, statistical testing of the differences using a T-test and finally a regression and cluster analysis. We performed all methods using the JASP program.

## Results

The following table provides an analysis of the number of companies in the engineering industry in crisis. The crisis is represented by bankruptcy, restructuring or liquidation. Most companies in crisis were in 2015 and the least at 53 in 2019, which was the last year analysed. In 2019, based on available data, we determined that 271 companies recorded significant debts. The records of debts in engineering companies are mainly related to the non-payment of taxes and insurance premiums, tax liabilities, liabilities to employees and liabilities from the supplier-customer relationship. The aim of the first part of the research was to answer the question whether enterprises of the Slovak engineering industry are creating a crisis situation through insolvency, profit making and bad management decisions in the observed period. The results are described in Table 1.

**Table 1:** An analysis of the number of companies in the engineering industry.

Category of crisis / year	2015	2016	2017	2018	2019
Number of enterprises	1909	1972	2037	2106	2102
Bankruptcies, restructuring, liquidations	145	126	97	79	53
Existence of debts	318	324	307	306	271

The next part of our research of the financial situation in engineering companies in Slovakia is a statistical testing of the significance of the relationships between the analysed variables. We will verify the normality of the distribution by means of the Shapiro-Wilk test (Table 2). At the level of significance α we determine the hypotheses of the test:

H_0_: the selected accounting items of engineering companies in Slovakia correspond to the normal distribution,
H_1_: the selected accounting items of engineering companies in Slovakia do not correspond to the normal distribution.

**Table 2:** Results of the Shapiro-Wilk test.

Descriptive Statistics											
-	stocks	property	short—term receivables	total equity	equity	profit after tax	short-term liabilities	sales revenue	profit or loss from economic activity	profit or loss from financial activity	profit before tax
Shapiro-Wilk	0.202	0.127	0.125	0.121	0.073	0.174	0.114	0.114	0.154	0.136	0.150
P-value of Shapiro-Wilk	< .001	< .001	< .001	< .001	< .001	< .001	< .001	< .001	< .001	< .001	< .001

By comparing the value of Sig. (p-value) from the previous table with the determined level of significance α we can state that for all items we do not achieve a normal distribution of variables. Based on previous testing of the normality of the distribution of selected accounting items, we will use the non-parametric Spearman correlation coefficient, which can be found in Appendix B, Table A1 in S1 Appendix, for parameters with a normal distribution within correlation coefficients.

At the level of significance α, all selected indicators of financial statements of engineering companies in Slovakia are statistically significant. For coefficients marked with an asterisk, the p value (p-value) is less than 0.001 and the values of the correlation coefficients are statistically significant (i.e. they are informative). In the analysis of the results, we can state that we achieved direct high dependencies on the assets and indicators, assets and equity, profit or loss from economic activity, profit after tax, profit before tax and profit from economic activity. It is true that some connections are logically related to each other and so their dependence is proven. The table is in the appendix to the paper.

One of the partial objectives of this contribution is to determine whether there are statistically significant differences between the indicators of financial performance of engineering companies between the individual years analysed. For the purposes of the analysis, we choose 3 key indicators: the first of them is the EBITDA indicator, which is based on the relationship (Profit before tax + interest + depreciation) / Sales [45].

Other variables are indicators based on methods of predicting the financial situation of companies. Model Index 05 and Altman’s index are among the most recognized and most used models in our conditions, i.e. they are adequate for use in the conditions of Slovak engineering companies. For the Altman index, we start with the formula:

$$\begin{array}{c} Z=3.3\times EBIT/Assets+1\times Sales/Assets+0.6\times Market\;value\;of\;equity/Book\;value\;of\\ debt+1.4\times Retained\;earnings/Assets+1.2\times Net\;working\;capital/Assets \end{array}$$

And based on the value that the company achieves, we classify the company in the group either as safe, grey or crisis zone [46].

Another model on the basis of which we can predict the financial situation of companies is the prediction model Index 05. Index 05 allows us to make a comprehensive conclusion about the performance of the company. The form of the equation for calculating the model values is as follows [47]:

$$\begin{array}{c} IN05=0.13\;*\;assets/debt+0.04\;*\;EBIT/interest\;expense+3.97\;*\;EBIT/total\;assets+\\ 0.21\;*\;sales/total\;assets+0.09\;*\;current\;assets/current\;liabilities \end{array}$$

The calculated values of the index 05 then divide the companies into individual intervals and on the basis of these calculations, it is possible with a high probability to predict the future development in the company [48]:

- IN05 ϵ (- ∞; 0.9˃ with a probability of 77% companies are bankrupt,
- IN05 ϵ (0.9; 1.6) the company is located in the grey zone, it is not possible to determine with sufficient probability in which direction it will develop in the future,
- IN05 ϵ <1.6; ∞) a creditworthy company, a company with an 83% probability of creating value.

The next part of the paper will be devoted to the analysis of the statistical significance of differences in the values of the above-mentioned financial indicators (Table 3). Before that, however, we present inductive statistics of selected indicators for the period between 2019 to 2015. Based on the median values of engineering companies, we can state that the achieved values are included in the group of creditworthy companies. Within the Altman Z-score, the median is in most cases on the border between a company that has a good financial and economic situation and is considered healthy versus a company that has an uncertain financial situation.

**Table 3:** Descriptive statistics of the statistical significance of differences in the values.

Descriptive Statistics															
-	EBITDA 2019	Altman Z score 2019	INDEX 05 2019	EBITDA 2018	Altman Z score 2018	INDEX 05 2018	EBITDA 2017	Altman Z score 2017	INDEX 05 2017	EBITDA 2016	Altman Z score 2016	INDEX 05 2016	EBITDA 2015	Altman Z score 2015	INDEX 05 2015
**Valid**	1990	1965	1910	1989	1949	1896	1932	1895	1875	1859	1816	1799	1792	1784	1760
**Missing**	114	139	194	115	155	208	172	209	229	245	288	305	312	320	344
**Mean**	270324.72	0.737	40.076	254606.55	1.203	245.046	234162.24	0.768	21.693	255813.16	-5.858	5.050	266257.72	-4.812	167.411
**Median**	10556.500	2.798	1.461	9938.000	2.596	1.385	10486.000	2.678	1.473	9867.000	2.568	1.358	9273.500	2.617	1.381
**Std. D**	2.292e +6	169.451	971.214	1.967e +6	173.809	10373.010	1.892e +6	172.626	619.012	2.066e +6	169.337	226.563	1.878e +6	182.345	6040.381
**Skewness**	18.756	-20.144	33.932	18.079	-16.759	43.527	14.570	-15.985	37.699	19.022	-28.248	3.071	15.630	-33.017	41.548
**Std. Error of Skewness**	0.055	0.055	0.056	0.055	0.055	0.056	0.056	0.056	0.057	0.057	0.057	0.058	0.058	0.058	0.058
**Kurtosis**	447.418	852.882	1216.139	435.648	838.629	1895.084	371.540	907.957	1537.245	440.600	912.552	264.153	300.887	1267.440	1736.574
**Std. Error of Kurtosis**	0.110	0.110	0.112	0.110	0.111	0.112	0.111	0.112	0.113	0.113	0.115	0.115	0.116	0.116	0.117

We will verify the normality of the distribution by means of the Shapiro-Wilk test (Table 4). At the level of significance α we determine the hypotheses of the test:

H_0_: the selected financial indicators of engineering companies in Slovakia correspond to the normal distribution,
H_1_: the selected financial indicators of engineering companies in Slovakia do not correspond to the normal distribution.

**Table 4:** Descriptive statistics of the Shapiro-Wilk test.

Descriptive Statistics															
-	EBITDA 2019	Altman Z score 2019	INDEX 05 2019	EBITDA 2018	Altman Z score 2018	INDEX 05 2018	EBITDA 2017	Altman Z score 2017	INDEX 05 2017	EBITDA 2016	Altman Z score 2016	INDEX 05 2016	EBITDA 2015	Altman Z score 2015	INDEX 05 2015
**Valid**	1990	1965	1910	1989	1949	1896	1932	1895	1875	1859	1816	1799	1792	1784	1760
**Shapiro-Wilk**	0.108	0.059	0.020	0.130	0.047	0.008	0.133	0.043	0.025	0.109	0.040	0.087	0.129	0.044	0.011
**P-value of Shapiro-Wilk**	< .001	< .001	< .001	< .001	< .001	< .001	< .001	< .001	< .001	< .001	< .001	< .001	< .001	< .001	< .001

**Fig 1:**
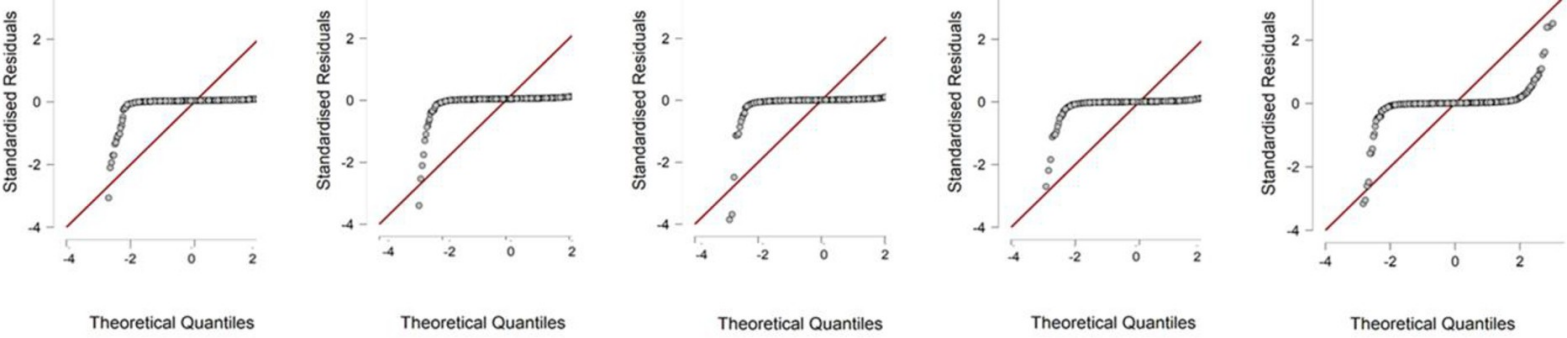
Altman Z score for the years between 2019–2015.

**Fig 2:**
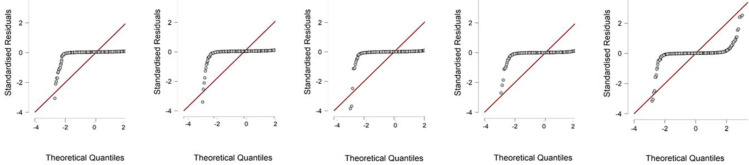
Index 05 for the years between 2019–2015.

By comparing the value of Sig. (p-value) from the previous table with the specified level of significance α we can say that for the indicators EBITDA, Altman Z score and Index 05 we reject the null hypothesis of normal distribution and state that individual indicators of financial performance of engineering companies for the period between 2019–2015 do not have a normal distribution. We can observe interesting results in the field of graphical representation of the results of prediction models using a Q-Q graph, which, among other things, allows us to assess the distribution of data. Within Altman’s Z score, we can say that engineering companies do not achieve a normal distribution, as we see in the graph (Fig 1). The Index 05 indicator reaches a similar value (Fig 2). We select the sample Q-Q plots for years.

For the sake of being complete, we also offer an analysis of relationships using the Spearman correlation coefficient in Appendix B—Table A2 in S1 Appendix, which shows that for some variables there is a statistically significant relationship and for others there is not where the p-value is less than the alpha level (0.001) and the values of correlation coefficients are statistically significant. In other relationships, the p-value of the correlation coefficients is greater than 0.001 and thus the correlation coefficients have no significance. In other relationships, the p-value of the correlation coefficients is greater than 0.001 and thus the correlation coefficients have no significance. We cannot judge whether there is a correlation between them or not. We can state that in all statistically significant pairs there is a direct dependence.

The test of normality (the table is in the appendix B in S1 Appendix to the paper) shows that in the research for some indicators that do not meet the condition of normality of the distribution, we cannot use the parametric test (t-test). In the t-test (Tables 5–7) we test the agreement of two average values of indicators between successive years (4 pairs for each indicator) at the level of significance α (0.05) and determine the following hypotheses of the test:

H_0_: there are no statistically significant differences between the values of the indicator between years.
H_1_: there are statistically significant differences between the values of the indicator between years.

**Table 5:** Paired samples T-Test of EBITDA.

Paired Samples T-Test				
Measure 1	Measure 2	t	df	p
**EBITDA 2019**	EBITDA 2018	-0.091	1882	0.928
**EBITDA 2018**	EBITDA 2017	0.228	1836	0.820
**EBITDA 2017**	EBITDA 2016	0.232	1766	0.817
**EBITDA 2016**	EBITDA 2015	-0.295	1702	0.768

**Table 6:** Paired samples T-Test of INDEX 05.

Paired Samples T-Test				
Measure 1	Measure 2	t	df	p
**INDEX 05 2019**	INDEX 05 2018	-0.940	1717	0.347
**INDEX 05 2018**	INDEX 05 2017	0.941	1745	0.347
**INDEX 05 2017**	INDEX 05 2016	1.169	1714	0.242
**INDEX 05 2016**	INDEX 05 2015	-1.118	1661	0.264

**Table 7:** Paired samples T-Test Altman Z score.

Paired Samples T-Test				
Measure 1	Measure 2	t	df	p
**Altman Z score 2019**	Altman Z score 2018	-0.068	1817	0.946
**Altman Z score 2018**	Altman Z score 2017	0.062	1811	0.950
**Altman Z score 2017**	Altman Z score 2016	1.043	1748	0.297
**Altman Z score 2016**	Altman Z score 2015	-0.226	1696	0.821

By comparing the value of Sig. (p-value) from the previous table with the specified level of significance α (0.001), we can state that for all indicators of financial performance of companies, the p-value is higher than the specified level of significance. We can state that the difference between the values of indicators between the individual years of enterprises in the engineering industry is statistically significant because the p-value is higher than α (0.05). We do not reject hypothesis H_0_.

Based on the values of financial indicators, which represent the representative characteristics of engineering companies, we decided to perform the clustering using the k-means method and came to the conclusion that we divided the 1248 companies that have been continuously operating for five years into the following groups of clusters (Table 8).

**Table 8:** Cluster information.

Cluster Information										
Cluster	1	2	3	4	5	6	7	8	9	10
**Size**	6	3	1	2	1212	1	1	1	6	15
**Explained proportion within-cluster heterogeneity**	0.133	0.019	0.000	5.658	0.615	0.000	0.000	0.000	0.115	0.119
**Within sum of squares**	1082.63	155.563	0.000	0.462	5016.09	0.000	0.000	0.000	934.993	967.211
**Silhouette score**	-0.015	0.316	0.000	0.956	0.856	0.000	0.000	0.000	0.050	0.216
**Centroid EBITDA 2019**	0.192	-0.065	-0.122	21.676	-0.035	0.444	-0.136	-0.135	-0.165	-0.093
**Centroid Altman Z score 2019**	0.022	-0.010	0.067	-0.014	-0.002	-0.014	1.448	0.242	-0.015	0.008
**Centroid INDEX 05 2019**	-0.037	-0.041	-0.033	0.096	-0.028	34.788	0.118	-0.016	-0.047	-0.039
**Centroid EBITDA 2018**	-0.091	-0.125	6.844	-0.126	-0.026	-0.127	-0.126	-0.129	-0.112	1.823
**Centroid Altman Z score 2018**	0.036	-0.338	-0.016	0.043	-0.026	-0.030	-0.039	32.687	-0.017	0.008
**Centroid INDEX 05 2018**	-0.029	-0.030	35.295	-0.029	-0.028	-0.029	-0.029	0.123	-0.029	-0.028
**Centroid EBITDA 2017**	10.628	-0.031	1.468	-0.132	-0.052	-0.164	-0.132	-0.142	-0.101	-0.071
**Centroid Altman Z score 2017**	-0.010	-0.078	-0.022	0.012	-0.026	-0.025	0.081	31.549	-0.002	-0.006
**Centroid INDEX 05 2017**	5.715	-0.036	-0.037	-0.034	-0.030	-0.038	-0.029	2.826	-0.036	-0.035
**Centroid EBITDA 2016**	-0.014	-0.129	0.007	-0.126	0.000	-0.152	0.067	-0.119	0.292	-0.075
**Centroid Altman Z score 2016**	0.051	-18.655	0.042	0.039	0.045	0.010	0.065	0.024	0.082	0.060
**Centroid INDEX 05 2016**	-0.022	-0.351	0.390	-0.046	-0.043	-0.054	-0.036	-0.040	8.855	-0.023
**Centroid EBITDA 2015**	-0.170	-0.179	-0.028	-0.166	-0.092	-0.183	1.373	-0.166	-0.131	7.521
**Centroid Altman Z score 2015**	0.040	0.045	0.141	0.044	0.043	0.002	0.044	0.065	-8.861	0.052
**Centroid INDEX 05 2015**	-0.032	-0.032	-0.029	0.053	-0.028	-0.032	35.183	-0.032	-0.049	-0.029

*Note*. The between sum of squares of the 10-cluster model is 10548.04.

*Note*. The Total sum of squares of the 10-cluster model is 18705.

The most numerous is cluster no. 5, which includes 1212 engineering companies, that makes up more than 97% of all engineering companies. Other clusters have significantly fewer companies (some only one company). We assume that this is due to the extreme values of indicators in the area of financial health prediction. Within the largest cluster, the values of the prediction models are in negative numbers, which may demonstrate their financial difficulties and there is a tendency to have poor financial results for these companies.

Another part of our research is the interpretation of the results of the dependence between the achieved EBIDTDA values and the results of the prediction models Z score and Index 05. The explanatory variable is EBITDA and the explained variables are the indicators of health prediction of engineering companies. The output of regression and correlation analysis consists of three parts (Table 9): the first part is the output of correlation analysis, the second part is the ANOVA output, where we test the suitability of the model used. The third part is the output of regression analysis. We performed statistical testing for each analysed year separately.

**Table 9:** Model Summary—EBITDA 2019.

**Model Summary—EBITDA 2019**				
**Model**	**R**	**R^2^**	**Adjusted R^2^**	**RMSE**
**H₀**	0.000	0.000	0.000	2.345e +6
**H₁**	0.132	0.018	0.017	2.325e +6

**ANOVA**						
**Model**	**Sum of Squares**	**df**	**Mean Square**	**F**	**p**	
**H₁**	Regression		1.832e +14	2	9.160e +13	16.943	< .001
**-**	Residual	1.026e +16	1897	5.406e +12		
**-**	Total	1.044e +16	1899		
**Coefficients**						
**Model**		**Unstandardized**	**Standard Error**	**Standardized**	**t**	**p**
**H₀**	(Intercept)	283053.161	53789.059		5.262	< .001
**H₁**	(Intercept)	270407.728	53426.147		5.061	< .001
**-**	INDEX 05 2019	318.995	54.799	0.132	5.821	< .001
**-**	Altman Z score 2019	-53.586	513.068	-0.002	-0.104	0.917

The first part of the Regression Statistics output is the result related to correlation analysis. The value of R (correlation coefficient) is equal to 0.132. In our example, there is a low degree of tightness when it comes to the relationship between the EBIDTA indicator and the values of the Z score and Index 05. The value of R Square is the value of the coefficient of determination; it is a value of 0.018. This value after multiplying 100 indicates that the chosen regression function explains the variability of the EBITDA indicator to about 18%, the other part represents unexplained variability, the influence of random factors and other unspecified influences. We achieve a similarly low percentage in the years between 2018–2015. For this reason, we will analyse the impact on EBITDA according to other explanatory variables. ANOVA testing showed that the model was chosen correctly as the p value at the F test <0.001.

In the next analysis (Tables 10–12), we focused on the relationship between EBITDA as the explanatory variable while inventories and short-term receivables were the explanatory variable. The value of R Square was at the level of 0.71 and the resulting regression function explains the variability to 71%. The hypothesis states the opposite. The F test was used to evaluate this statement. Significance F = <0.001, so based on statistical analysis, hypotheses H0 can be rejected. The model that was identified was chosen correctly.

**Table 10:** Model Summary–EBITDA.

Model Summary—EBITDA				
Model	R	R^2^	Adjusted R^2^	RMSE
**H₀**	0.000	0.000	0.000	3.044e +6
**H₁**	0.844	0.713	0.713	1.632e +6

**Table 11:** ANOVA.

ANOVA						
Model		Sum of Squares	df	Mean Square	F	p
**H₁**	Regression	7.375e +15	2	3.688e +15	1384.888	< .001
**-**	Residual	2.966e +15	1114	2.663e +12	-	-
**-**	Total	1.034e +16	1116	-	-	-

*Note*. The intercept model is omitted, as no meaningful information can be shown.

**Table 12:** Coefficients.

Coefficients						
Model		Unstandardized	Standard Error	Standardized	t	p
**H₀**	(Intercept)	459132.846	91082.131		5.041	< .001
**H₁**	(Intercept)	-110149.636	50015.235		-2.202	0.028
**-**	Stocks	0.538	0.018	0.678	29.819	< .001
**-**	Short–term receivables	0.096	0.010	0.216	9.498	< .001

Subsequently, we analysed the relationship between EBITDA as the explanatory variable while sales revenue and equity were the explanatory variable (Tables 13–15). The value of R Square is at the level of 0.88 and the resulting regression function explains the variability to 88%, the other part is unexplained variability. The F test is used to evaluate this statement. Significance F = <0.001, so based on statistical analysis, hypotheses H0 can be rejected. The model that was identified was chosen correctly.

**Table 13:** Model Summary–EBITDA.

Model Summary—EBITDA				
Model	R	R^2^	Adjusted R^2^	RMSE
**H₀**	0.000	0.000	0.000	2.391e +6
**H₁**	0.938	0.880	0.880	828905.125

**Table 14:** ANOVA.

ANOVA						
Model		Sum of Squares	df	Mean Square	F	p
**H₁**	Regression	9.180e +15	2	4.590e +15	6680.485	< .001
**-**	Residual	1.253e +15	1823	6.871e +11	-	-
**-**	Total	1.043e +16	1825	-	-	-

*Note*. The intercept model is omitted, as no meaningful information can be shown.

**Table 15:** Coefficients.

Coefficients						
Model		Unstandardized	Standard Error	Standardized	t	p
**H₀**	(Intercept)	294693.682	55952.007	-	5.267	< .001
**H₁**	(Intercept)	-43018.174	19618.846	-	-2.193	0.028
**-**	sales revenue	0.065	0.002	0.657	36.864	< .001
**-**	equity	0.082	0.005	0.304	17.063	< .001

The regression function has the form y = -43018.174 + 0.065 x1 + 0.082 x2. The P-value (Values) will be used to evaluate these statements. The P-value for Intercept H_0_ (locating constant) is <0.001. This suggests that the locating constant is statistically insignificant but for H_1_ it is 0.028> 0.001, which indicates statistical significance. The P-value for the regression coefficient b1 is <0.001, which confirms the significance of this coefficient. The P-value for the regression coefficient b2 is <0.001, which also confirms the significance of this coefficient.

## Discussion

Based on the performed analyses, we can draw several conclusions. The first is the fact that a small number (2.5% of companies in 2019) had to resolve their financial situation through crisis management—liquidation, bankruptcy or restructuring. During the analysed period this was a declining trend, which was a positive development in the field of bankruptcy and restructuring. In the correlation analyses, we found that selected indicators of the state and flow from the balance sheet as well as the profit and loss statement showed high direct dependencies—but some are based on the logic of their continuity (e.g. sales and profit). Subsequently, we calculated the values of the predictive indicators Index In and Z score as well as the indicator EBIDTA, which we considered to be key indicators in this research. Using testing normality, we again proceeded to the analysis of the dependence between these indicators for the observed period between 2015–2019. Using a paired t-test, we determined the statistical significance of the differences between the key indicators in the engineering industry and found that there was a statistical significance between the differences in the indicators for the following years in the industry. By implementing the cluster, we came to the conclusion that the cluster was the most numerous and included 1212 engineering companies, which represented more than 97% of all engineering companies. We can say that these companies achieved similar results by three selected methods: the Altman score, the index and EBIDTA for the period between 2015 to 2019. The last part of our research was the analysis of factors that affected individual key indicators. We performed this analysis using a regression function.

In the discussion, we would like to state the development of insolvency in selected sectors of engineering 33, 28 and 29, as general engineering and automotive represent the largest parts of the engineering sector in Slovakia (other sectors such as the production of ships and locomotives have a negligible part of the Slovak engineering industry). The result is a table (Table 16) that contains the years between 2016 to 2019 from the point of view of insolvency of engineering companies. It is characteristic of insolvency that the primary insolvency is> 1 and the secondary insolvency is <1. The table below summarizes the insolvency results for enterprises divided into lower quartile, median, upper quartile and average, and divides the companies in terms of assets and turnover.

**Table 16:** The average of the financial indicator of insolvency.

***33—Repair and installation of machines and apparatus–INSOLVENCY***						***Property in mil*. *€***			***Turnover in mil*. *€***		
***2016***	***2017***	***2018***	***2019***	***St*. *char*.**	***Unit***	***< 1*.*6***	***1*.*6–5*.*0***	***> 1*.*6***	***< 0*.*8***	***0*.*8–3*.*3***	***> 3*.*3***
0,73	0,73	0,66	0,63	LQ	coef.	0,65	0,32	0,35	0,65	0,61	0,36
1,47	1,58	1,48	1,48	Me	coef.	1,59	0,65	1,05	1,71	1,07	0,79
3,58	5,03	4,25	4,53	UQ	coef.	5,09	1,24	1,90	5,93	2,26	1,86
29,13	12,50	38,25	14,49	Avg	coef.	15,51	1,41	5,66	17,05	3,21	1,22
***28—Manufacture of machinery and equipment–INSOLVENCY***						***Property in mil*. *€***			***Turnover in mil*. *€***		
***2016***	***2017***	***2018***	***2019***	***St*. *char*.**	***Unit***	***< 1*.*6***	***1*.*6–5*.*0***	***> 1*.*6***	***< 0*.*8***	***0*.*8–3*.*3***	***> 3*.*3***
0,91	0,89	0,80	0,88	LQ	coef.	0,97	0,76	0,76	0,97	0,83	0,77
1,83	1,80	1,61	1,90	Me	coef.	2,13	1,25	1,30	2,58	1,40	1,28
5,50	6,24	5,35	6,42	UQ	coef.	13,12	3,08	2,58	20,99	2,70	2,42
17,97	18,23	19,73	24,93	Avg	coef.	31,91	7,26	2,22	38,72	2,86	2,16
***29—Manufacture of motor vehicles*, *semi-trailers and trailers–INSOLVENCY***						***Property in mil*. *€***			***Turnover in mil*. *€***		
***2016***	***2017***	***2018***	***2019***	***St*. *char*.**	***Unit***	***< 1*.*6***	***1*.*6–5*.*0***	***> 1*.*6***	***< 0*.*8***	***0*.*8–3*.*3***	***> 3*.*3***
0,95	1,02	0,96	0,74	LQ	coef.	0,67	1,14	0,82	0,71	0,66	0,82
2,30	3,02	2,68	2,11	Me	coef.	2,62	2,51	1,69	3,25	0,92	1,71
5,86	13,09	10,58	15,93	UQ	coef.	73,07	3,57	3,40	155,76	2,51	3,13
201,30	22,10	23,90	105,02	Avg	coef.	151,04	52,56	2,71	169,85	5,37	2,49

The analysis shows that engineering companies with low turnover or assets perform worse in terms of insolvency than companies with higher turnover or assets.

Overall, we can state that the engineering industry in Slovakia from the point of view of financial analysis during the observed period appears to be improving. Significant shifts may occur after the publication of financial results of companies in connection with COVID-19, as there was a decrease in production and a decrease in orders in the sector not only from Slovakia but especially from abroad. In this effort to analyse and compare the results before and after the crisis, we will certainly continue to work on in the future.

Enterprises of the Slovak engineering industry are achieving a crisis in the observed period. Based on the analysis we performed and using various indicators, we can say that the crisis of companies in the engineering industry in Slovakia is in a slight downturn. The numbers of companies that have debts or companies that have declared bankruptcy, restructuring, liquidation or bankruptcy, is lower from year to year. We cannot predict the consequences for the engineering industry of the crisis caused by COVID-19. However, several industries such as metallurgy did not decline but responded to the strong recovery of the second half of 2020, when they were also helped by the regulation of cheap Asian imports in the form of tariffs and maintained double-digit year-on-year growth in December 2020 (more than 12%). At the end of the year, the engineering industry was joined by the engineering industry (more than 17%), which still showed a year-on-year decline in production in November. Of course, the increase in percentages is related to exports [49].

Regarding the hypotheses, hypothesis 1: There are statistically significant differences between the values of indicators of the crisis of engineering companies between the analysed years. Based on the performed t-tests, we can state that for all financial performance indicators (Altman’s Z-score, IN05 index and EBIDTA indicator) of engineering companies, the value of p is higher than the specified level of significance. We can state that the difference between the values of indicators between individual years of enterprises in the engineering industry is statistically significant. Statistical significance is manifested primarily by the fact that the engineering industry in Slovakia has long-term stable results and in the area of indicators of the company’s crisis, values are improving, which is documented not only by research results but also other sources that contain other relevant outputs in this area [50–53].

Hypothesis 2: Within the indicators of the corporate crisis—Altman Z-score and the IN05 index, there are statistically significant factors that affect the value of these indicators. Using the performed regression analysis and ANOVA, we tried to determine which key factors have an impact on the EBITDA indicator, which we can perceive as a partial tool for assessing the corporate crisis. From the analyses performed, we can summarize the fact that the selected variables affect and some do not affect EBIDTA values, as we mentioned in the results section. When it comes to future development, it would be appropriate to focus on other factors that are being investigated by several authors. The approaches are focused on the analysis of several indicators of financial performance of companies, which are represented by indicators of asset rentability and return on equity. Many of them focus on the analysis of factors from the supplier-customer point of view. Others examine the impact of profitability and technological assets on a company’s financial performance [54–56]. However, in several studies we can observe a departure from traditional factors that monitor the impact on the corporate crisis. These authors solve the problem rather than achieving the values of financial indicators through management techniques and methods, which will not indicate a crisis, for example, through total quality management [57].

## Conclusions

The role of financial managers of engineering companies is to maintain the good financial results they showed in the past. In the current period, this will be relatively difficult because the loss of orders will certainly lead to a reduction in sales and thus will lead to a reduction in the achieved profit of companies. Financial managers must look for the optimization of financial processes and obtain savings, e.g. in the field of marketing, reduction of representation costs or even the remuneration of employees with the result of cost savings. Time that we can consider "empty" due to the non-utilization of production capacities can be used by engineering companies and from the saved resources they have achieved (confirmed by their financial analysis for the period between 2015–2019) they could implement innovations and investments in their company. Of course, with investments it is necessary to secure a sufficiently large source of finance—it can be equity or foreign capital, or the financial resources that are provided with the help of subsidy bodies of the European Union and resources provided by institutions in Slovakia [58].

Ensuring the desired level during the crisis situation of companies in engineering can be achieved with several tools, which brings crisis management. There are two forms in which we can solve crisis within engineering companies. The first of these is the bankruptcy of the company, when the company’s assets are sold and the creditors are satisfied with the proceeds of the sale. The second option is restructuring. In this form, the company is rehabilitated and the company is not liquidated as in bankruptcy. The result of the restructuring is a plan which provides for a change in the ownership, organizational, legal and other structures of the company in order to overcome the crisis in the area of financing, insolvency and liquidity crisis [59,60].

The approaches of several authors have led to a unified concept of solving crisis situations in companies. Entries in this area contain solutions that should lead companies to correctly identify and find solutions in crisis management in the company [61]. It seems unambiguous to perceive the issue of the crisis in the company depending on the life cycle of the company and to adapt the appropriate crisis management tools. There will also be authors approaching crisis management in companies through the preparation of crisis situations and the practice of behaviour not only of management but also of the overall company. This tool serves as prevention against a possible crisis. By being prepared for a crisis, employees know how to handle it better [62]. The last method we would like to mention is the risk and vulnerability assessment method, which can be used to diagnose a liquidity, profitability or insolvency problem over time. On the basis of an early warning, the company can carry out preliminary remediation procedures and processes to avert the consequences of the crisis, as stated by several authors [63].

Our findings have important implications for business owners / SMEs, scientists and policy makers from emerging economies with similar financial systems. In terms of practical implications, this study suggests that corporate bankruptcy is improving in all achieved indicators (since the outbreak of the COVID-19 pandemic, we have not been able to determine bankruptcy as the data is not disclosed). In this context, SMEs should monitor the development of indicators such as predictive models, traditional models—especially liquidity and solvency.

For academics, our study provides evidence of the numbers of companies that have debts or companies that have declared bankruptcy, restructuring, liquidation or bankruptcy, while at the same time the numbers of companies are lower from year to year. Our contribution therefore contributes to the insufficient literature on the role of bankruptcies in the engineering industry in Slovakia. The statistical significance is manifested primarily by the fact that the engineering industry in Slovakia has long-term stable results and in the area of indicators of the corporate crisis, the values are improving.

There are limitations to our study that we should be aware of. It is related to the lack of information on the issue of the corporate crisis during the ongoing COVID -19 pandemic.

In the practice of companies, the solution of the corporate crisis is a key issue from the point of view of financial indicators and they should also monitor it according to the methods we mentioned in this section. Such restrictions have in the implementation of trials, were insufficient when it comes to data and information resources. For this reason, our planned analysis of the liquidity crisis in the engineering sector was significantly limited. We hope that this issue will be discussed in the future. The conclusions drawn from these empirical analyses are limited by the data on which the results are based and cannot be generalized to different categories of companies from other countries. As for future research, it would be interesting to extend the analysis to other developing countries (e.g. the Czech Republic is comparable to Slovakia in a similar development of the economy in the area of GDP and a similar accounting system) and to other time periods. However, this approach reduces the bias of the small class and allows comparisons across countries and over time. Also, new phenomena in economics should be considered, such as the effects of the shared economy, digital services and the increasing use of virtual currencies as well as changes in taxation of transnational companies with potential impact on the financial management of companies.

## Supporting information

S1 Appendix (DOCX)
