# Supplementary material for: Factors predicting companies’ crisis in the engineering industry from the point of view of financial analysis
Source: PLoS One. 2022 Feb 18;17(2):e0264016. doi: 10.1371/journal.pone.0264016 (PMC9116989; doi:10.1371/journal.pone.0264016)
Supplement: S1 Appendix — (DOCX) [file pone.0264016.s001.docx]

**Appendix A**

The contribution included enterprises from the following sectors according to SK NACE:

Specifically, we dealt with the engineering sector and according to SK NACE include: Installation of industrial machinery [33200], Installation of industrial machinery and equipment [33200], Repair and maintenance of ships [33150], Repair and maintenance of ships and boats [33150], Repair and maintenance other means of transport [33170], Repair and maintenance of aircraft and spacecraft [33160], Repair of metal structures [33110], Repair of other equipment [33190], Repair of machinery [33120], Construction of ships and floating structures [30110], Construction of recreational and sports boats [30120], Manufacture of metal forming machinery [28410], Manufacture of honeycomb machinery [28960], Manufacture of mining machinery [28920], Manufacture of bicycles [30920], Manufacture of bicycles and wheelchairs [30920], Manufacture of lifting and handling equipment [28220], Manufacture of non-domestic refrigeration and ventilation equipment [28250], Manufacture of refrigeration equipment [28250], Manufacture of other pumps [28130], Manufacture of other pumps and compressors [28130], Manufacture of other machinery wefts and valves [28140], Manufacture of office machinery and equipment (except computers and peripheral equipment) [28230], Manufacture of bearings [28150], Manufacture of bearings, gears, gearing and driving elements [28150], Manufacture of motorcycles [30910], Manufacture engines and turbines [28110], Manufacture of engines and turbines, except aircraft, vehicle and cycle engines [28110], Manufacture of other special machinery [28990], Manufacture of other general machinery [28290], Manufacture of other machine tools [28490], Manufacture of other special purpose machinery [28990], Manufacture of other general purpose machinery [28290], Manufacture of agricultural machinery [28300], Manufacture of food machinery [28930], Manufacture of power-driven hand tools [ 28240], Manufacture of ovens, furnaces and furnace burners [28210], Manufacture of machinery for metal working [28410], Manufacture of machinery for food, beverage and tobacco processing [28930], Manufacture of plastics and rubber machinery [28960], Manufacture of machinery for mining, quarrying and construction [28920], Manufacture of machinery for metallurgy [28910], Manufacture of agricultural and forestry machinery [28300], Manufacture of machinery for textile, apparel and leather production [28940], Manufacture of railway locomotives and rolling stock [30200].

**Appendix B**

Table A1. Spearman correlation coefficient for variables

| **Variable** | |  | | **property** | | **stocks** | | **short - term receivables** | | **total equity** | | **equity** | | **profit after tax** | | **short-term liabilities** | | **sales revenue** | | **profit or loss from economic activity** | | **profit or loss from financial activity** | | **profit before tax** | |  |
| --- | --- | --- | --- | --- | --- | --- | --- | --- | --- | --- | --- | --- | --- | --- | --- | --- | --- | --- | --- | --- | --- | --- | --- | --- | --- | --- |
| 1. property |  | n |  | — |  |  |  |  |  |  |  |  |  |  |  |  |  |  |  |  |  |  |  |  |  |  |
|  |  | Spearman's rho |  | — |  |  |  |  |  |  |  |  |  |  |  |  |  |  |  |  |  |  |  |  |  |  |
|  |  | p-value |  | — |  |  |  |  |  |  |  |  |  |  |  |  |  |  |  |  |  |  |  |  |  |  |
| 2. stocks |  | n |  | 1133 |  | — |  |  |  |  |  |  |  |  |  |  |  |  |  |  |  |  |  |  |  |  |
|  |  | Spearman's rho |  | 0.751 | *** | — |  |  |  |  |  |  |  |  |  |  |  |  |  |  |  |  |  |  |  |  |
|  |  | p-value |  | < .001 |  | — |  |  |  |  |  |  |  |  |  |  |  |  |  |  |  |  |  |  |  |  |
| 3. short - term receivables |  | n |  | 1878 |  | 1119 |  | — |  |  |  |  |  |  |  |  |  |  |  |  |  |  |  |  |  |  |
|  |  | Spearman's rho |  | 0.869 | *** | 0.642 | *** | — |  |  |  |  |  |  |  |  |  |  |  |  |  |  |  |  |  |  |
|  |  | p-value |  | < .001 |  | < .001 |  | — |  |  |  |  |  |  |  |  |  |  |  |  |  |  |  |  |  |  |
| 4. total equity |  | n |  | 2062 |  | 1133 |  | 1877 |  | — |  |  |  |  |  |  |  |  |  |  |  |  |  |  |  |  |
|  |  | Spearman's rho |  | 0.799 | *** | 0.587 | *** | 0.706 | *** | — |  |  |  |  |  |  |  |  |  |  |  |  |  |  |  |  |
|  |  | p-value |  | < .001 |  | < .001 |  | < .001 |  | — |  |  |  |  |  |  |  |  |  |  |  |  |  |  |  |  |
| 5. equity |  | n |  | 2045 |  | 1128 |  | 1865 |  | 2053 |  | — |  |  |  |  |  |  |  |  |  |  |  |  |  |  |
|  |  | Spearman's rho |  | 0.542 | *** | 0.530 | *** | 0.476 | *** | 0.458 | *** | — |  |  |  |  |  |  |  |  |  |  |  |  |  |  |
|  |  | p-value |  | < .001 |  | < .001 |  | < .001 |  | < .001 |  | — |  |  |  |  |  |  |  |  |  |  |  |  |  |  |
| 6. profit after tax |  | n |  | 2018 |  | 1127 |  | 1865 |  | 2025 |  | 2007 |  | — |  |  |  |  |  |  |  |  |  |  |  |  |
|  |  | Spearman's rho |  | 0.494 | *** | 0.256 | *** | 0.479 | *** | 0.609 | *** | 0.166 | *** | — |  |  |  |  |  |  |  |  |  |  |  |  |
|  |  | p-value |  | < .001 |  | < .001 |  | < .001 |  | < .001 |  | < .001 |  | — |  |  |  |  |  |  |  |  |  |  |  |  |
| 7. short-term liabilities |  | n |  | 1990 |  | 1132 |  | 1862 |  | 1996 |  | 1978 |  | 1977 |  | — |  |  |  |  |  |  |  |  |  |  |
|  |  | Spearman's rho |  | 0.844 | *** | 0.740 | *** | 0.772 | *** | 0.502 | *** | 0.480 | *** | 0.329 | *** | — |  |  |  |  |  |  |  |  |  |  |
|  |  | p-value |  | < .001 |  | < .001 |  | < .001 |  | < .001 |  | < .001 |  | < .001 |  | — |  |  |  |  |  |  |  |  |  |  |
| 8. sales revenue |  | n |  | 1826 |  | 1090 |  | 1746 |  | 1826 |  | 1811 |  | 1821 |  | 1820 |  | — |  |  |  |  |  |  |  |  |
|  |  | Spearman's rho |  | 0.843 | *** | 0.675 | *** | 0.816 | *** | 0.724 | *** | 0.488 | *** | 0.546 | *** | 0.763 | *** | — |  |  |  |  |  |  |  |  |
|  |  | p-value |  | < .001 |  | < .001 |  | < .001 |  | < .001 |  | < .001 |  | < .001 |  | < .001 |  | — |  |  |  |  |  |  |  |  |
| 9. profit or loss from economic activity |  | n |  | 1987 |  | 1130 |  | 1857 |  | 1990 |  | 1973 |  | 1985 |  | 1958 |  | 1826 |  | — |  |  |  |  |  |  |
|  |  | Spearman's rho |  | 0.544 | *** | 0.310 | *** | 0.524 | *** | 0.633 | *** | 0.201 | *** | 0.972 | *** | 0.383 | *** | 0.597 | *** | — |  |  |  |  |  |  |
|  |  | p-value |  | < .001 |  | < .001 |  | < .001 |  | < .001 |  | < .001 |  | < .001 |  | < .001 |  | < .001 |  | — |  |  |  |  |  |  |
| 10. profit or loss from financial activity |  | n |  | 1914 |  | 1118 |  | 1802 |  | 1918 |  | 1903 |  | 1914 |  | 1886 |  | 1768 |  | 1899 |  | — |  |  |  |  |
|  |  | Spearman's rho |  | -0.603 | *** | -0.497 | *** | -0.533 | *** | -0.431 | *** | -0.340 | *** | -0.243 | *** | -0.554 | *** | -0.594 | *** | -0.337 | *** | — |  |  |  |  |
|  |  | p-value |  | < .001 |  | < .001 |  | < .001 |  | < .001 |  | < .001 |  | < .001 |  | < .001 |  | < .001 |  | < .001 |  | — |  |  |  |  |
| 11. profit before tax |  | n |  | 2004 |  | 1130 |  | 1859 |  | 2009 |  | 1991 |  | 2004 |  | 1965 |  | 1826 |  | 1990 |  | 1918 |  | — |  |  |
|  |  | Spearman's rho |  | 0.518 | *** | 0.276 | *** | 0.504 | *** | 0.628 | *** | 0.183 | *** | 0.991 | *** | 0.352 | *** | 0.571 | *** | 0.980 | *** | -0.256 | *** | — |  |  |
|  |  | p-value |  | < .001 |  | < .001 |  | < .001 |  | < .001 |  | < .001 |  | < .001 |  | < .001 |  | < .001 |  | < .001 |  | < .001 |  | — |  |  |

Table A2. Spearman's Correlations coefficient

| **Spearman's Correlations** | | | | | | | | | | | | | | | | | | | | | | | | | | | | | | | |
| --- | --- | --- | --- | --- | --- | --- | --- | --- | --- | --- | --- | --- | --- | --- | --- | --- | --- | --- | --- | --- | --- | --- | --- | --- | --- | --- | --- | --- | --- | --- | --- |
| **Variable** | |  | | **EBITDA 2019** | | **Altman Z score 2019** | | **INDEX 05 2019** | | **EBITDA 2018** | | **Altman Z score 2018** | | **INDEX 05 2018** | | **EBITDA 2017** | | **Altman Z score 2017** | | **INDEX 05 2017** | | **EBITDA 2016** | | **Altman Z score 2016** | | **INDEX 05 2016** | | **EBITDA 2015** | | **Altman Z score 2015** | |
| 1. EBITDA 2019 |  | Spearman's rho |  | — |  |  |  |  |  |  |  |  |  |  |  |  |  |  |  |  |  |  |  |  |  |  |  |  |  |  |  |
|  |  | p-value |  | — |  |  |  |  |  |  |  |  |  |  |  |  |  |  |  |  |  |  |  |  |  |  |  |  |  |  |  |
| 2. Altman Z score 2019 |  | Spearman's rho |  | 0.265 |  | — |  |  |  |  |  |  |  |  |  |  |  |  |  |  |  |  |  |  |  |  |  |  |  |  |  |
|  |  | p-value |  | < .001 |  | — |  |  |  |  |  |  |  |  |  |  |  |  |  |  |  |  |  |  |  |  |  |  |  |  |  |
| 3. INDEX 05 2019 |  | Spearman's rho |  | 0.461 |  | 0.782 |  | — |  |  |  |  |  |  |  |  |  |  |  |  |  |  |  |  |  |  |  |  |  |  |  |
|  |  | p-value |  | < .001 |  | < .001 |  | — |  |  |  |  |  |  |  |  |  |  |  |  |  |  |  |  |  |  |  |  |  |  |  |
| 4. EBITDA 2018 |  | Spearman's rho |  | -0.024 |  | 0.020 |  | 0.015 |  | — |  |  |  |  |  |  |  |  |  |  |  |  |  |  |  |  |  |  |  |  |  |
|  |  | p-value |  | 0.302 |  | 0.391 |  | 0.517 |  | — |  |  |  |  |  |  |  |  |  |  |  |  |  |  |  |  |  |  |  |  |  |
| 5. Altman Z score 2018 |  | Spearman's rho |  | -0.026 |  | -0.012 |  | 0.008 |  | 0.253 |  | — |  |  |  |  |  |  |  |  |  |  |  |  |  |  |  |  |  |  |  |
|  |  | p-value |  | 0.271 |  | 0.607 |  | 0.743 |  | < .001 |  | — |  |  |  |  |  |  |  |  |  |  |  |  |  |  |  |  |  |  |  |
| 6. INDEX 05 2018 |  | Spearman's rho |  | -0.018 |  | -0.005 |  | 0.007 |  | 0.446 |  | 0.768 |  | — |  |  |  |  |  |  |  |  |  |  |  |  |  |  |  |  |  |
|  |  | p-value |  | 0.438 |  | 0.841 |  | 0.763 |  | < .001 |  | < .001 |  | — |  |  |  |  |  |  |  |  |  |  |  |  |  |  |  |  |  |
| 7. EBITDA 2017 |  | Spearman's rho |  | -0.041 |  | -0.040 |  | -0.059 |  | 0.065 |  | -0.032 |  | -0.026 |  | — |  |  |  |  |  |  |  |  |  |  |  |  |  |  |  |
|  |  | p-value |  | 0.078 |  | 0.087 |  | 0.013 |  | 0.005 |  | 0.174 |  | 0.275 |  | — |  |  |  |  |  |  |  |  |  |  |  |  |  |  |  |
| 8. Altman Z score 2017 |  | Spearman's rho |  | -0.039 |  | -0.008 |  | -0.046 |  | -0.033 |  | -0.007 |  | 0.004 |  | 0.261 |  | — |  |  |  |  |  |  |  |  |  |  |  |  |  |
|  |  | p-value |  | 0.102 |  | 0.749 |  | 0.058 |  | 0.163 |  | 0.751 |  | 0.881 |  | < .001 |  | — |  |  |  |  |  |  |  |  |  |  |  |  |  |
| 9. INDEX 05 2017 |  | Spearman's rho |  | -0.019 |  | -0.018 |  | -0.055 |  | -0.033 |  | -0.035 |  | -0.035 |  | 0.460 |  | 0.766 |  | — |  |  |  |  |  |  |  |  |  |  |  |
|  |  | p-value |  | 0.429 |  | 0.439 |  | 0.022 |  | 0.164 |  | 0.139 |  | 0.143 |  | < .001 |  | < .001 |  | — |  |  |  |  |  |  |  |  |  |  |  |
| 10. EBITDA 2016 |  | Spearman's rho |  | -0.057 |  | -0.026 |  | -0.038 |  | 0.051 |  | -0.003 |  | 0.016 |  | 0.078 |  | 0.007 |  | -0.006 |  | — |  |  |  |  |  |  |  |  |  |
|  |  | p-value |  | 0.017 |  | 0.277 |  | 0.117 |  | 0.032 |  | 0.896 |  | 0.503 |  | < .001 |  | 0.776 |  | 0.793 |  | — |  |  |  |  |  |  |  |  |  |
| 11. Altman Z score 2016 |  | Spearman's rho |  | -0.019 |  | -0.018 |  | -0.045 |  | -0.019 |  | 0.014 |  | 0.007 |  | -0.032 |  | 0.056 |  | 0.038 |  | 0.312 |  | — |  |  |  |  |  |  |  |
|  |  | p-value |  | 0.430 |  | 0.468 |  | 0.070 |  | 0.431 |  | 0.569 |  | 0.776 |  | 0.187 |  | 0.019 |  | 0.110 |  | < .001 |  | — |  |  |  |  |  |  |  |
| 12. INDEX 05 2016 |  | Spearman's rho |  | -0.046 |  | -0.006 |  | -0.034 |  | -0.017 |  | 0.021 |  | 0.027 |  | -0.007 |  | 0.029 |  | 0.023 |  | 0.502 |  | 0.765 |  | — |  |  |  |  |  |
|  |  | p-value |  | 0.056 |  | 0.809 |  | 0.167 |  | 0.475 |  | 0.392 |  | 0.276 |  | 0.783 |  | 0.221 |  | 0.336 |  | < .001 |  | < .001 |  | — |  |  |  |  |  |
| 13. EBITDA 2015 |  | Spearman's rho |  | 0.008 |  | -0.023 |  | 0.009 |  | 0.105 |  | 0.017 |  | 0.032 |  | 0.020 |  | -0.035 |  | -0.014 |  | 0.115 |  | 0.017 |  | 0.039 |  | — |  |  |  |
|  |  | p-value |  | 0.734 |  | 0.341 |  | 0.715 |  | < .001 |  | 0.488 |  | 0.190 |  | 0.412 |  | 0.146 |  | 0.571 |  | < .001 |  | 0.496 |  | 0.108 |  | — |  |  |  |
| 14. Altman Z score 2015 |  | Spearman's rho |  | -0.012 |  | -0.016 |  | -0.025 |  | 0.034 |  | 0.038 |  | 0.034 |  | -0.042 |  | 0.002 |  | -0.007 |  | 0.004 |  | 0.048 |  | 0.007 |  | 0.307 |  | — |  |
|  |  | p-value |  | 0.628 |  | 0.503 |  | 0.313 |  | 0.167 |  | 0.121 |  | 0.164 |  | 0.084 |  | 0.929 |  | 0.766 |  | 0.881 |  | 0.050 |  | 0.769 |  | < .001 |  | — |  |
|  | | | | | | | | | | | | | | | | | | | | | | | | | | | | | | | |
